# The complex role of immune cells in antigen presentation and regulation of T-cell responses in hepatocellular carcinoma: progress, challenges, and future directions

**DOI:** 10.3389/fimmu.2024.1483834

**Published:** 2024-10-22

**Authors:** Jianbo Ning, Yutao Wang, Zijia Tao

**Affiliations:** ^1^ The Fourth Clinical College, China Medical University, Shenyang, China; ^2^ Department of Urology, Peking Union Medical College Hospital, Chinese Academy of Medical Sciences and Peking Union Medical College, Beijing, China; ^3^ Department of Interventional Radiology, the First Hospital of China Medical University, Shenyang, China

**Keywords:** hepatocellular carcinoma, dendritic cells, natural killer cells, CTLA-4, PD-1/PD-L1

## Abstract

Hepatocellular carcinoma (HCC) is a prevalent form of liver cancer that poses significant challenges regarding morbidity and mortality rates. In the context of HCC, immune cells play a vital role, especially concerning the presentation of antigens. This review explores the intricate interactions among immune cells within HCC, focusing on their functions in antigen presentation and the modulation of T-cell responses. We begin by summarizing the strategies that HCC uses to escape immune recognition, emphasizing the delicate equilibrium between immune surveillance and evasion. Next, we investigate the specific functions of various types of immune cells, including dendritic cells, natural killer (NK) cells, and CD8+ T cells, in the process of antigen presentation. We also examine the impact of immune checkpoints, such as cytotoxic T-lymphocyte-associated protein 4 (CTLA-4) and the pathways involving programmed cell death protein 1 (PD-1) and programmed death ligand 1 (PD-L1), on antigen presentation, while taking into account the clinical significance of checkpoint inhibitors. The review further emphasizes the importance of immune-based therapies, including cancer vaccines and CAR-T cell therapy, in improving antigen presentation. In conclusion, we encapsulate the latest advancements in research, propose future avenues for exploration, and stress the importance of innovative technologies and customized treatment strategies. By thoroughly analyzing the interactions of immune cells throughout the antigen presentation process in HCC, this review provides an up-to-date perspective on the field, setting the stage for new therapeutic approaches.

## 1 Introduction

Hepatocellular carcinoma (HCC), the predominant type of liver cancer, poses a considerable challenge to global health, illustrated by rising incidence figures and concerning mortality rates (1–3). The complex relationships between cancerous cells and the host’s immune system are crucial in influencing the development and progression of HCC. Understanding how immune cells participate in the antigen presentation process related to HCC is vital, as this insight is fundamental to improving our grasp of tumor immunology and formulating effective therapeutic strategies (4). This review seeks to explore the complex interactions between immune cells and HCC, emphasizing their functions in antigen presentation and the regulation of T cell responses (5). HCC is frequently associated with chronic liver conditions, such as viral hepatitis, alcoholic liver disease, and non-alcoholic fatty liver disease (5, 6). These risk factors lead to a microenvironment that fosters immune dysregulation, allowing tumor cells to escape immune detection and grow (7, 8).

The immune system’s ability to recognize and destroy abnormal cells is fundamental to cancer progression. In this context, the process of antigen presentation acts as a critical checkpoint (9, 10). Antigen-presenting cells (APCs), including dendritic cells (DCs) and macrophages, play a vital role in the capture of tumor-specific antigens and their subsequent delivery to CD8+ cytotoxic T cells. This process initiates a cascade of immune responses aimed at targeting cancerous cells. Nevertheless, the complex mechanisms linked to antigen presentation in HCC and their influence on the anti-tumor immune response remain subjects of ongoing research.

Understanding the immune dynamics related to HCC antigen presentation carries important therapeutic implications. While immune checkpoint inhibitors have achieved impressive results across various types of cancer, their effectiveness in HCC has been limited. This discovery suggests that gaining a deeper insight into the immune landscape within the microenvironment of HCC is essential for creating improved immunotherapy approaches (11, 12). Additionally, creating personalized treatment modalities, such as cancer vaccines and adoptive T cell therapies, relies on a clear understanding of the mechanisms of antigen presentation (13, 14).

As investigations reveal the complex interactions among immune cells and HCC, it is essential to present a thorough summary of the current understanding. This review aims to consolidate existing research to illuminate the varied functions of immune cells in relation to antigen presentation within HCC. By exploring the detailed communication among immune cells, tumor cells, and the tumor microenvironment, we seek to clarify the elements that contribute to immune evasion and identify potential pathways for enhancing anti-tumor immune responses. Through this analysis, the review enhances the overall comprehension of tumor immunology and establishes a foundation for developing innovative and focused immunotherapeutic approaches targeting HCC.

### 1.1 Immune escape mechanism of hepatocellular carcinoma

HCC is recognized as the main liver cancer that often arises in the context of ongoing liver inflammation, particularly associated with conditions such as viral hepatitis and liver damage from alcohol (15–17). The management of immune surveillance plays a crucial role in identifying and eliminating cancerous cells. Nevertheless, HCC has skillfully developed various strategies to avoid immune detection, promoting its unrestricted growth and advancement (18). An essential factor contributing to the immune evasion observed in HCC is the increased expression of inhibitory immune checkpoint proteins. Among these, programmed death ligand 1 (PD-L1) stands out prominently, alongside cytotoxic T-lymphocyte-associated protein 4 (CTLA-4) (19–21). These molecules interact with their corresponding receptors on T cells, hindering their activation and subsequent effector functions, thereby weakening the immune response aimed at cancerous cells.

**Figure 1:**
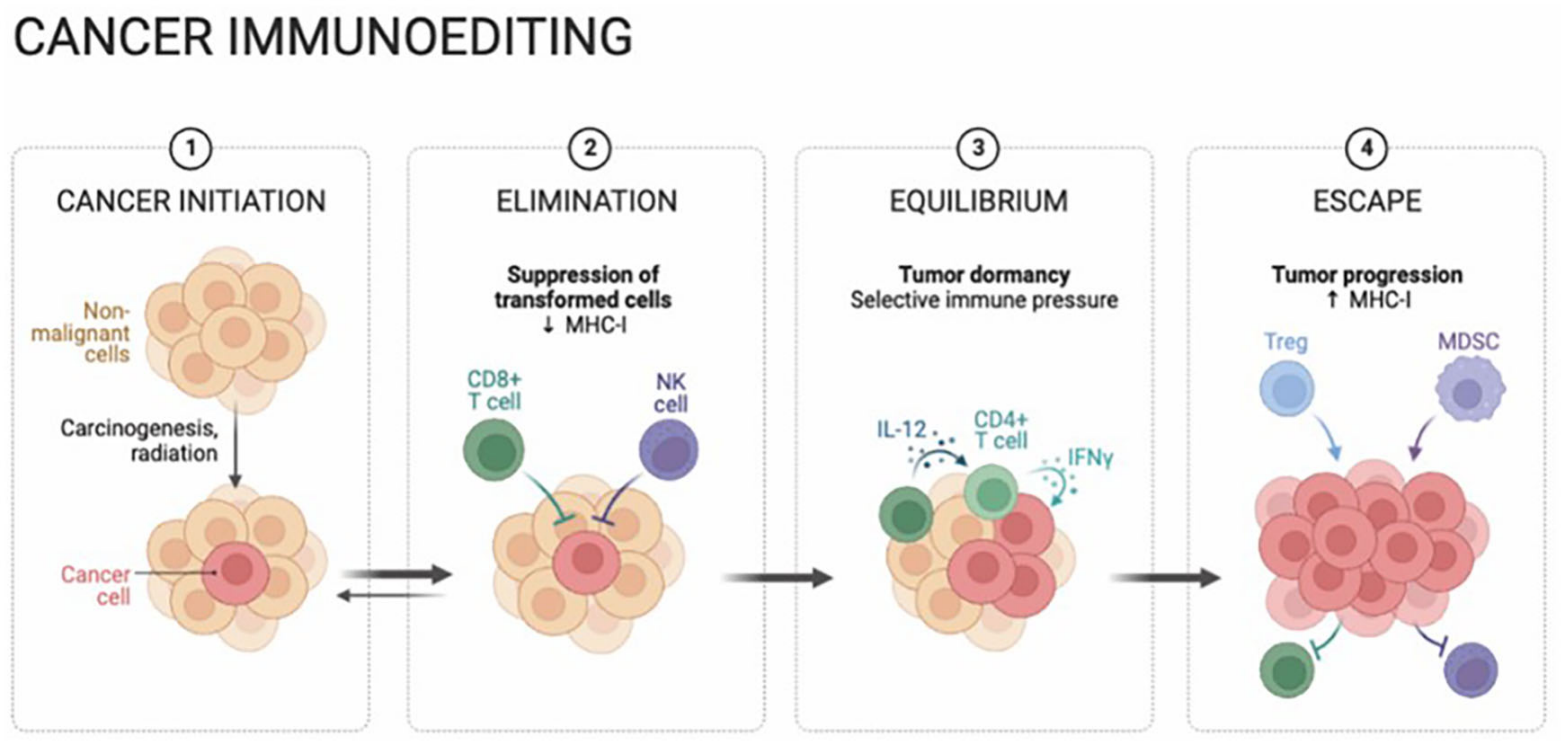
Hepatocellular carcinoma (HCC) cancer immunoediting is a dynamic and multifaceted process, comprising the stages of cancer initiation, elimination, equilibrium, and eventual escape. Immune surveillance initially destroys nascent cells, followed by immune equilibrium controlling growth. Over time, HCC evades detection, leading to progression. Understanding these stages reveals the complex immune-HCC relationship, emphasizing therapeutic potential.

Furthermore, the microenvironment surrounding tumors in hepatocellular carcinoma (HCC) frequently exhibits a suppressive immune profile, largely as a result of the buildup of regulatory T cells (Tregs) and myeloid-derived suppressor cells (MDSCs) (22–24). This aggregation of cells leads to the suppression of effector T cells and natural killer (NK) cells, ultimately undermining the immune response directed at malignant cells (25, 26). At the same time, HCC cells display a tendency to release a variety of immunosuppressive cytokines and chemokines, particularly transforming growth factor-beta (TGF-β) and interleukin-10 (IL-10), contributing to the overall reduction of anti-tumoral immune responses (**Figure 1**).

Alterations in both genetic and epigenetic factors observed in HCC cells contribute to immune evasion, as demonstrated by changes in human leukocyte antigen (HLA) expression and modifications in mechanisms for antigen presentation (27–29). These irregularities enable HCC cells to avoid detection and destruction by cytotoxic T cells, crafting a sophisticated evasion strategy. Recently, clinical studies have highlighted the effectiveness of immunotherapeutic approaches aimed at these immune escape routes. In these initiatives, immune checkpoint blockers like anti-programmed cell death protein 1 (PD-1) and anti-PD-L1 antibodies have demonstrated effectiveness in certain groups of HCC patients by re-energizing the inhibited immune response directed towards the tumor. Furthermore, investigating combination therapies targeting various immune evasion pathways shows potential in enhancing the effectiveness of treatment option.

### 1.2 Interaction between immune cells and tumor microenvironment

The complex interactions between various groups of immune cells and the tumor microenvironment (TME) in HCC represent a dynamic and multifaceted mechanism that significantly influences the progression of the disease (11, 30). A broad spectrum of research has uncovered important elements of this relationship, emphasizing the sophisticated ways in which distinct immune cell populations interact and communicate within the TME. Tumor-associated macrophages (TAMs) are key players in the immune milieu, predominantly exhibiting a pro-tumoral M2 phenotype in the context of HCC. This behavior fosters both tumor development and neovascularization by releasing factors such as vascular endothelial growth factor (VEGF) and IL-10 (31, 32). Tregs, another critical aspect of the immune response, build up in the TME and considerably influence antitumor immunity by effectively inhibiting cytotoxic T cells and establishing an immunosuppressive environment (33). Concurrently, MDSCs play a pivotal role in promoting immune evasion mechanisms, as they reduce T cell functionality and stimulate angiogenesis, further complicating the already intricate landscape of immune escape within the TME (34).

Additionally, the complex interactions of immune checkpoints in the HCC microenvironment trigger a series of sophisticated molecular exchanges that shape the trajectory of antitumor immune responses. Importantly, PD-1 found on T cells, when engaging with its corresponding ligand PD-L1 on tumor cells, induces a state of immune exhaustion, undermining the processes of effective tumor recognition and elimination (35, 36). In a similar vein, CTLA-4 inhibits the activation of antitumor T cell responses through its proficient suppression of costimulatory signals (37, 38). These diverse interactions collectively create a complex landscape dependent on a fragile balance between immune activation and inhibition, a balance that is significantly disrupted in the context of HCC.

Deepening our understanding of these complex interactions offers considerable potential for identifying new therapeutic avenues for HCC. Targeted strategies that modulate immune cell dynamics and interrupt the detailed network of immune evasion mechanisms may prove effective in halting tumor progression and enhancing antitumor immune responses. The rapid progress of breakthroughs in immunotherapy, including immune checkpoint blockers and adoptive T cell treatments, underscores the growing dynamism within this field. Furthermore, the integration of cutting-edge methodologies, including single-cell genomics and spatial transcriptomics, has the potential to reveal previously unexplored complexities within the immune environment of HCC (39). These revelations could lead to the creation of precision immunotherapeutic strategies specifically designed to address the unique immunological context of individual HCC patients, signaling a new era in the management of this challenging malignancy.

### 1.3 Balance of immune surveillance and escape

The intricate equilibrium between immune monitoring and evasion significantly impacts the advancement of HCC. A range of research efforts supports the complex interactions between tumor cells and the immune system of the host. While immune surveillance conducts a complex process to recognize and eliminate cancerous cells, HCC employs various strategies to evade detection and destruction by the immune system. The mechanisms of immune evasion in HCC are illustrated by the heightened expression of inhibitory immune checkpoint proteins, notably characterized by increased levels of PD-L1 (40, 41). This orchestrated interaction with PD-1 on T cells leads to their exhaustion and results in a weakened antineoplastic effector response (42). The TME within HCC acts as an architect, shaping an environment conducive to immunosuppression, which is significantly influenced by the recruitment of regulatory Tregs and MDSCs. The consolidation of these immunosuppressive entities ultimately suppresses the functionality of effector immune cells. This delicate equilibrium is further influenced by fluctuating levels of pro-inflammatory cytokines, notably IL-6 and tumor necrosis factor-alpha (TNF-α), which play dual roles by enhancing antitumoral immunity while also promoting inflammation within the tumor microenvironment (43–45).

Therapeutic strategies designed to counteract these complex mechanisms include immune checkpoint inhibitors and adoptive T cell therapies. The primary aim of these therapeutic approaches is to restore balance, thereby enhancing immune responses against HCC cells. Immune checkpoint inhibitors, known for their ability to disrupt immune inhibitory interactions, represent a powerful front in overcoming the immune-suppressive barriers established by HCC. In conjunction with this, adoptive T cell therapies provide a means to rejuvenate the functionality of effector T cells, thereby revitalizing the immune response. Together, these therapeutic modalities hold transformative potential, envisioning a therapeutic landscape that favors a recalibrated immune response against HCC and, consequently, a more effective therapeutic trajectory.

## 2 Role of immune cells in HCC antigen presentation

**Figure 2:**
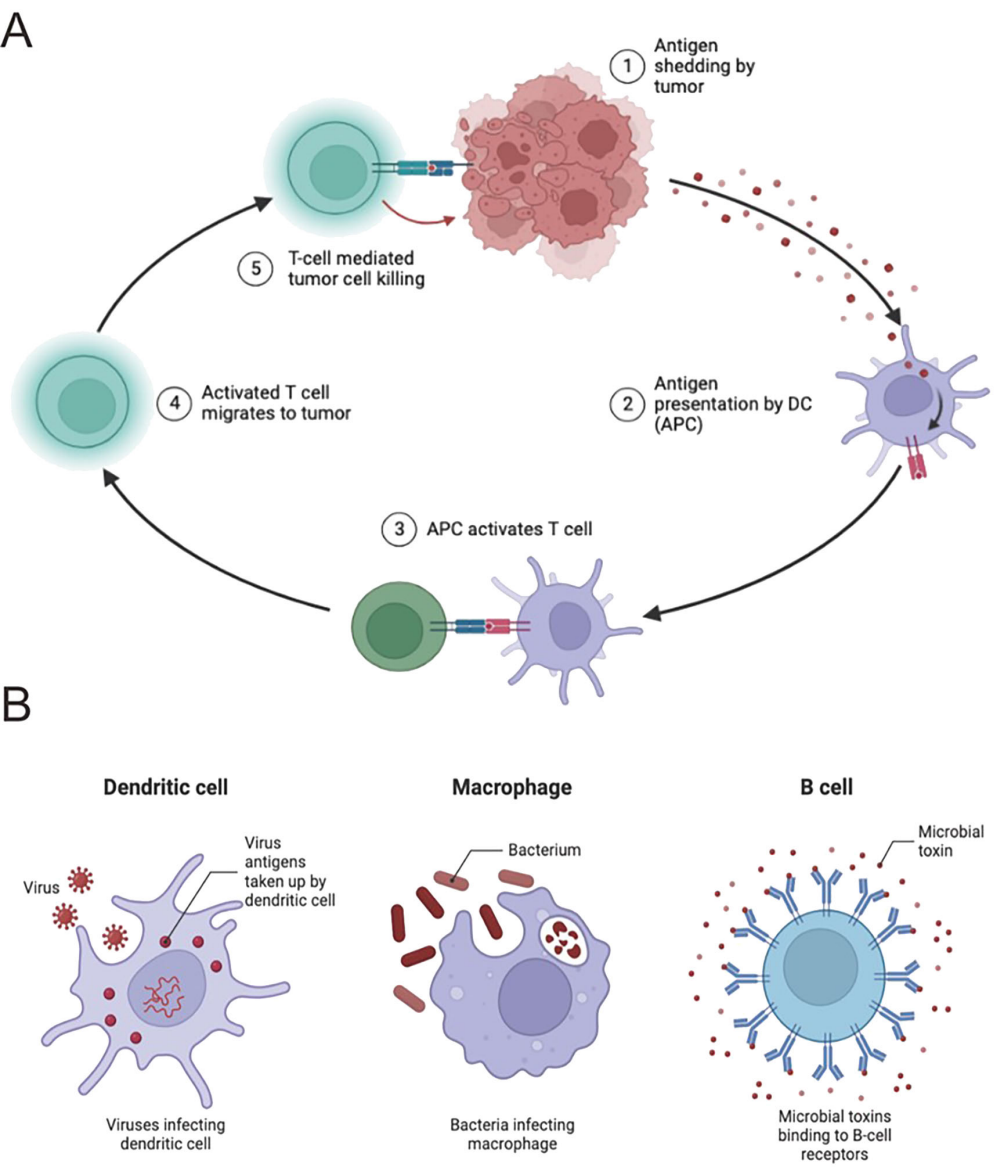
Immune cells play a pivotal role in HCC antigen presentation. **(A)** Antigen presentation by immune cells orchestrates anticancer responses. Dendritic cells (DCs) capture antigens and present to CD8+ T cells, with CD4+ T cells enhancing this process. HCC develops immune escape mechanisms, impairing antigen presentation. In-depth study may reveal new immunotherapy targets. **(B)** DCs capture and process antigens for presentation to CD8+ T cells. NK cells directly kill HCC cells. CD4+ T cells assist in antitumor immunity. HCC employs immune escape mechanisms; comprehensive understanding is crucial for treatment strategies.

In the realm of HCC, the coordination of immune cells is vital to the intricate process of presenting antigens, which is essential for triggering adaptive immune responses aimed at combating cancer cells. Dendritic cells (DCs) are particularly important in this immune response, as they adeptly capture antigens from tumor cells, process them, and present tumor-derived peptides on their surface within the major histocompatibility complex (MHC) framework (46, 47). This essential interaction between antigens and MHC molecules is recognized by CD4+ and CD8+ T cells, which in turn triggers a specific immune response against HCC (48–50). However, HCC can evade this immune response through various mechanisms that interfere with antigen presentation, leading to immune resistance. Notably, tumor-infiltrating myeloid cells, including DCs, undergo changes that render them immunosuppressive, diminishing their ability to present antigens effectively. Moreover, HCC cells themselves can modulate MHC expression or suppress antigen processing and presentation, enabling them to escape detection by T cells. Therapeutic strategies, such as immune checkpoint inhibitors, are designed to counter these inhibitory signals and enhance antigen presentation, aiming to elicit robust antitumor immune responses, which holds promise in the treatment of HCC (**Figure 2**).

As we delve deeper into this intricate environment, the expanding array of immune cell subsets within the HCC microenvironment significantly influences the dynamics of antigen presentation. Regulatory T cells (Tregs), known for their suppressive effects on immune responses, interact closely with antigen-presenting DCs, modulating the strength and effectiveness of immune activation (51) (**Figure 2**). MDSCs, characterized by their strong immunosuppressive properties, contribute to a complex interplay that dampens antigen presentation, creating a microenvironment that supports HCC progression. Understanding and targeting this complex interplay is crucial for developing new therapies that overcome immune suppression, enhance antigen presentation, and strengthen antitumor immune responses (52).

From a broader therapeutic perspective, the quest to enhance antigen presentation and reinvigorate immune responses is fraught with challenges. The inherent plasticity of the immune landscape, coupled with the complexity of the HCC microenvironment, complicates the development of effective interventions. While immune checkpoint inhibitors have shown promise, ongoing efforts must balance the enhancement of immune responses with the potential for immune-related adverse effects. The potential for combination therapies, including but not limited to immune checkpoint inhibitors, offers hope in dismantling the mechanisms of immune escape (53, 54). Identifying the most effective and lasting therapeutic approaches will require careful exploration through a multidisciplinary approach that integrates experimental findings with clinical observations.

### 2.1 Function and regulation of dendritic cells

DCs play a crucial role at the crossroads of the immune system, orchestrating key regulatory functions within both the innate and adaptive immune responses (55, 56). As specialized antigen-presenting cells, DCs are responsible for capturing, processing, and presenting antigens to T cells, thereby initiating and modulating a range of immune responses. In the complex environment of HCC, the essential role of DCs is evident in their ability to recognize tumor-derived antigens and initiate the priming of tumor-specific T cells (57, 58, 108). DCs utilize a variety of mechanisms, including phagocytosis, macropinocytosis, and receptor-mediated endocytosis, to capture tumor antigens. The antigens that have been captured are subsequently transformed into peptide fragments, which are displayed on the surface of DCs through major histocompatibility complex (MHC) molecules. This process of presenting antigens, aided by co-stimulatory signals from DCs, triggers the activation of naïve T cells, ultimately leading to the formation of tumor-specific cytotoxic CD8+ T cells and CD4+ T helper cells.

The functionality of DCs is closely tied to their maturation process, which is meticulously regulated. While immature DCs are highly efficient at capturing antigens, they have limited ability to stimulate T cells (59, 60). This changes when DCs are exposed to inflammatory signals, such as pathogen-associated molecular patterns (PAMPs) or danger-associated molecular patterns (DAMPs). These signals drive DCs into a mature state, characterized by increased surface expression of co-stimulatory molecules like CD80 and CD86, and the release of various cytokines. Maturation is critical for equipping DCs with the capacity to effectively prime T cells (61, 62). However, the tumor microenvironment in HCC can influence DC function, potentially leading to the development of tolerogenic DCs. These tolerogenic DCs exhibit reduced antigen presentation and diminished T cell activation, contributing to immune evasion by the tumor.

### 2.2 Role of natural killer cells

NK cells are a critical component of the innate immune defense, playing a vital role in the surveillance of HCC and other malignancies. These specialized lymphocytes have the unique ability to detect and eliminate target cells, including tumor cells, without requiring prior sensitization (63, 64). In HCC, NK cells are key players in antitumor immunity, primarily due to their capacity to recognize stress-induced ligands such as MHC class I-related chain A/B (MICA/B) and UL16-binding proteins (ULBPs) on the surface of cancer cells. The cytotoxic arsenal of activated NK cells, which includes perforin and granzymes, is instrumental in triggering apoptosis in tumor cells. Beyond direct cytotoxicity, NK cells also secrete immune-modulatory cytokines, notably interferon-gamma (IFN-γ), which amplify the immune response and contribute to a coordinated antitumor effect (65). Nonetheless, the function of NK cells in HCC is intricate and greatly influenced by the tumor microenvironment. The immunosuppressive nature of the HCC microenvironment can dampen NK cell activity through various mechanisms. A major factor is the upregulation of inhibitory ligands, such as PD-L1, on tumor cells, which interact with PD-1 receptors on NK cells, leading to functional exhaustion. Additionally, the presence of Tregs and MDSCs further suppresses NK cell function. To counteract these inhibitory influences and enhance NK cell activity in HCC, research is increasingly focused on innovative immunotherapies, including immune checkpoint inhibitors and adoptive NK cell therapies.

To summarize, NK cells have an essential yet intricate function in the immune response to HCC. While they contribute significantly to tumor surveillance and destruction, the tumor microenvironment presents formidable challenges that undermine their effectiveness. Advances in immunotherapy that harness the full potential of NK cells may offer new therapeutic avenues and transform treatment strategies for HCC.

### 2.3 CD8+ T cell function and activation

Cytotoxic T cells (CTLs), referred to as CD8+ T lymphocytes, play a crucial role in the immune system’s response to HCC and various other cancers (66). These cells are highly adept at recognizing and eliminating infected or transformed cells, making them crucial for maintaining immune surveillance. In the HCC microenvironment, their activation is initiated when their T cell receptors (TCRs) interact with tumor-associated antigens presented by major histocompatibility complex class I (MHC-I) molecules, typically facilitated by professional antigen-presenting cells such as dendritic cells (67). This interaction triggers the activation of CD8+ T cells, leading to their clonal expansion and differentiation into effector CTLs. These effector cells then migrate to the tumor site, where they deploy their cytotoxic arsenal, including perforin and granzymes, to induce apoptosis in cancer cells. Additionally, CD8+ T cells produce cytokines like IFN-γ, which not only enhance the antitumor immune response but also activate other immune cells, thereby contributing to the establishment of an immunogenic environment within the tumor microenvironment.

Despite their significant role, CD8+ T cells face numerous challenges within the HCC microenvironment. A major obstacle is the creation of an immunosuppressive environment, rich in soluble factors such as vascular endothelial growth factor (VEGF) and transforming growth factor-beta (TGF-β), which negatively impact CD8+ T cell activation and function (68). Moreover, HCC cells often downregulate MHC-I expression, rendering themselves less visible to CD8+ T cells and disrupting their ability to recognize and target malignant cells (69). To overcome these challenges, therapeutic strategies have been developed, focusing on immune checkpoints like the PD-1/PD-L1 axis (109). These approaches aim to block inhibitory pathways, thereby restoring the full functional capacity of CD8+ T cells and improving clinical outcomes for patients with HCC.

### 2.4 Other immune cell types contribute

Besides the main immune cell subsets mentioned earlier, a varied range of additional immune cell populations contributes significantly to the intricate immune reaction against HCC (70, 71). Neutrophils, for instance, exhibit a dual role depending on their polarization, functioning both in promoting tumor growth and in exerting antitumor effects. Some subsets of neutrophils contribute to inflammation and cancer progression, while others support antitumor immunity by enhancing T cell infiltration and activation. Mast cells also significantly influence the tumor microenvironment by releasing various mediators that promote angiogenesis and recruit other immune cells. Although present in lower numbers within the HCC microenvironment, B cells contribute to antitumor immunity through antibody production and antigen presentation. Recent research has emphasized the role of γδ T cells and innate lymphoid cells (ILCs) in regulating the immune response in HCC, which may impact tumor development and alter treatment results.

## 3 Regulation of immune checkpoint in HCC during antigen presentation

The control of immune checkpoints while presenting antigens is vital for maintaining a balance between immune activation and tolerance in the context of HCC (37, 72). The intricate relationship involving immune checkpoints like PD-1 and its ligand, PD-L1, plays a significant role in influencing the immune response directed at cancer cells (73). Within the HCC environment, inflammatory signals promote the increased expression of PD-L1 on tumor cells, which aids in forming an immunosuppressive microenvironment. This process involves the interaction between CD8+ T cells and antigen-presenting cells (APCs), including dendritic cells. The interaction between PD-1 on T cells and PD-L1 on APCs results in T cell fatigue, which diminishes their ability to produce cytokines and perform cytotoxic functions. This mechanism, while preventing excessive immune activity, also allows tumor cells to evade immune detection.

Findings from clinical research underscore the promise of immune checkpoint blockade as a treatment strategy. Employing anti-PD-1/PD-L1 antibodies represents a potentially effective method to rejuvenate fatigued CD8+ T cells, thus amplifying the immune response toward tumors in patients with HCC (74). As our understanding of the delicate interplay between immune checkpoints, antigen presentation, and immune evasion in HCC expands, strategies aimed at deciphering and modifying this complex relationship offer the potential for substantial progress in boosting the effectiveness of antitumor immunotherapy. The active modulation of immune checkpoints during the process of antigen presentation highlights their essential function in influencing the immune response against HCC. Approaches focused on modifying immune checkpoint interactions could significantly enhance antitumor immunity and improve clinical outcomes for HCC patients.

### 3.1 The role of CTLA-4 and PD-1/PD-L1 pathways

**Figure 3:**
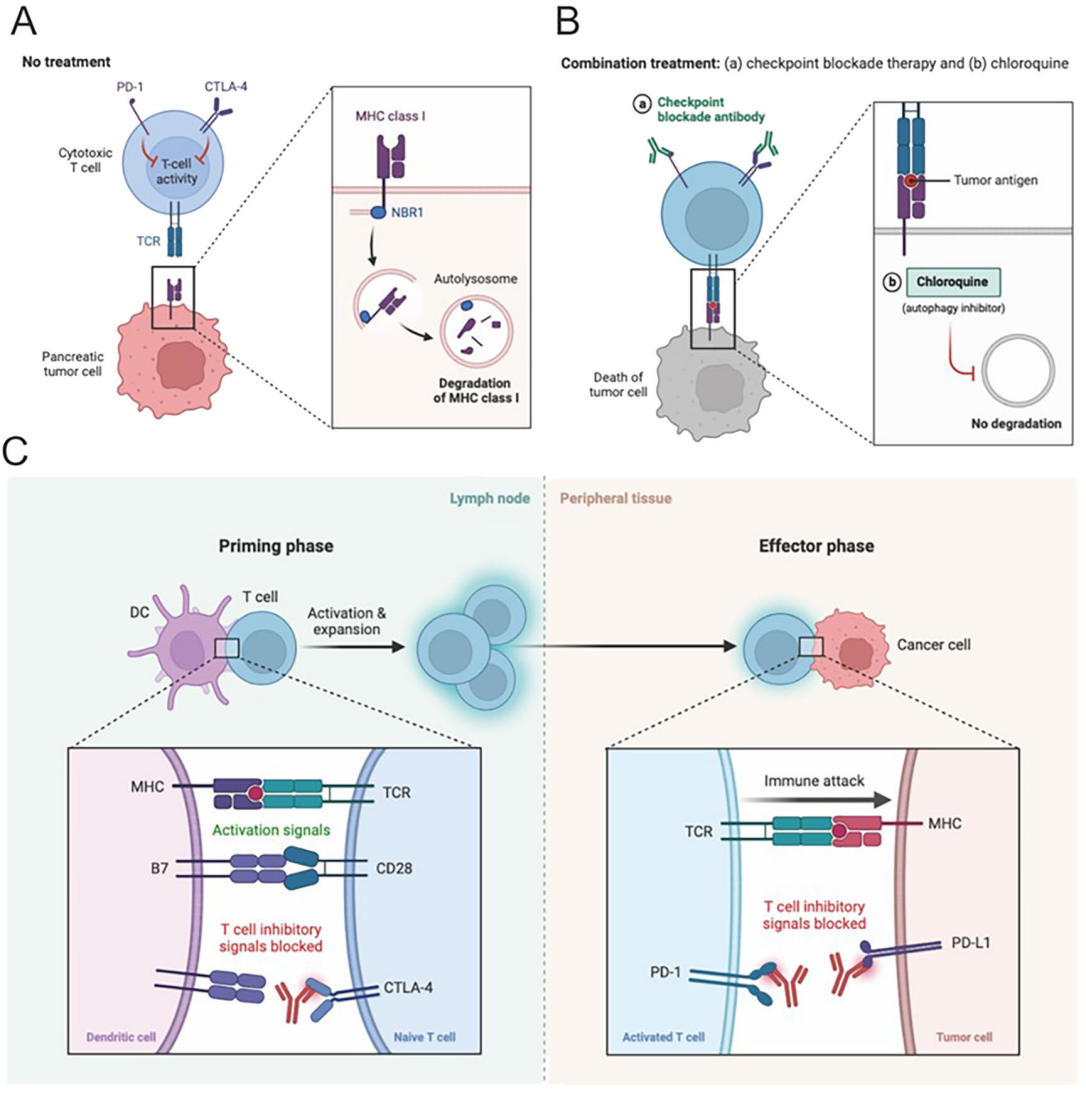
Combination therapies hold great potential for HCC. **(A, B)** Pancreatic cancer in the context of HCC often requires multifaceted treatment. The combination of immune checkpoint inhibitors with tyrosine kinase inhibitors or locoregional therapies is an attractive strategy, aiming to synergistically impede tumor growth, etc. This leverages complementary mechanisms of different treatments, laying the foundation for effective therapy. **(C)** Blocking CTLA-4 or PD-1 signaling is a promising approach in HCC immunotherapy. These checkpoints regulate T cell activity and are exploited by tumor cells to evade immune surveillance. Inhibitors targeting them release T cell responses. Clinical trials show encouraging results, potentially revolutionizing HCC treatment. Manipulating these pathways is a promising strategy to enhance immunity and improve outcomes.

The intricate involvement of the CTLA-4 and PD-1/PD-L1 pathways in HCC underscores their essential roles in regulating immune responses and significantly impacting tumor development (**Figure 3A**). CTLA-4, a key receptor expressed on activated T cells, competes with the CD28 receptor for binding to B7 ligands on antigen-presenting cells. By doing so, CTLA-4 functions as a negative regulator, effectively inhibiting T cell activation and acting as an immune checkpoint that prevents hyperactive immune responses, which could otherwise lead to autoimmune damage (75). In the context of HCC, CTLA-4 exerts its suppressive effects by curtailing the activity of tumor-infiltrating lymphocytes, thereby facilitating tumor immune evasion and contributing to the progression of the malignancy (**Figure 3**).

Conversely, the PD-1 pathway serves a unique function in preserving peripheral tolerance and inhibiting autoimmunity. PD-1 is expressed on the surface of activated T cells, B cells, and myeloid cells, and interacts with its ligands, PD-L1 and PD-L2, which are expressed on a variety of cell types, including both neoplastic and immune cells (76, 77). This interaction is particularly relevant in the microenvironment of HCC, where the binding of PD-1 on T cells to PD-L1 on tumor or immune cells creates an immunosuppressive environment that promotes T cell exhaustion. The fatigue reduces T cells’ capability to efficiently target cancer cells, thereby undermining the immune system’s effectiveness in regulating tumor development.

Clinical studies have provided compelling evidence for the therapeutic potential of targeting these immune checkpoints in HCC. The development and application of immune checkpoint inhibitors, such as ipilimumab, which targets CTLA-4, and nivolumab, which targets the PD-1/PD-L1 axis, have shown promising results in enhancing antitumor immune responses. These inhibitors work by reinvigorating exhausted T cells, thereby restoring their ability to fight against tumor cells and leading to improved survival rates among HCC patients. The success of these therapies has opened new avenues for the treatment of HCC and has underscored the critical importance of continued research into the mechanisms of immune checkpoint regulation.

### 3.2 Clinical application of immune checkpoint inhibitors

The clinical integration of immune checkpoint inhibitors has revolutionized the therapeutic landscape for HCC and various other malignancies (78). By strategically focusing on regulatory pathways like CTLA-4 and PD-1/PD-L1, these inhibitors activate the immune system’s natural ability to identify and destroy cancerous cells. In the context of HCC, these agents have shown significant promise. Notably, well-conducted clinical trials, including KEYNOTE-240 and CheckMate-040, have provided robust evidence of the efficacy and safety of immune checkpoint inhibitors like pembrolizumab and nivolumab in treating advanced HCC (79–81). These trials have demonstrated notable improvements in overall survival and sustained responses in a specific subset of patients. Combination therapies involving immune checkpoint inhibitors and other immunotherapies exhibit substantial potential in HCC. Evidence indicates that these combinations can synergistically enhance efficacy by activating the immune system through multiple pathways, thereby improving therapeutic outcomes (82, 83). As our understanding of HCC immunological mechanisms advances and new therapies continue to emerge, these combination strategies are likely to be further refined, offering improved survival benefits for HCC patients. The potential of combining immune checkpoint inhibitors with other therapeutic strategies, such as tyrosine kinase inhibitors and locoregional treatments, holds great promise for enhancing therapeutic outcomes (84). However, as this therapeutic approach continues to gain traction, several critical considerations must be addressed. Essential elements comprise the classification of patients according to significant clinical indicators, the discovery of predictive biomarkers, and the meticulous management of adverse immune-related effects. These aspects are vital for enhancing the clinical application of immune checkpoint inhibitors. The main challenges in implementing personalized immunotherapy for HCC include tumor heterogeneity, the complexity of immune escape mechanisms, and the lack of effective biomarkers for predicting therapeutic efficacy. Future studies should concentrate on a more thorough investigation of the immune evasion mechanisms in HCC, the identification of novel therapeutic targets and biomarkers, as well as the evaluation of the safety and effectiveness of new treatments via extensive clinical trials. The scientific community must continue to advance research efforts, focusing on strategies that effectively integrate immune checkpoint inhibitors with other treatments and identify prognostic markers. This ongoing research is essential to developing personalized treatment regimens tailored to the unique characteristics of individual patients.

## 4 Application of antitumor immunotherapy in enhancing antigen presentation

Antitumor immunotherapy signifies a groundbreaking method for treating cancer, improving the presentation of antigens and bolstering the immune system’s reaction to malignant cells. This method leverages the complex interactions among immune cells, tumor antigens, and the tumor microenvironment to thwart immune evasion and reinstate effective immune surveillance. In the context of HCC and various other malignancies, treatment approaches seek to enhance the pathways for antigen presentation, thereby promoting the activation of CTLs along with other essential immune cells.

A fundamental component of antitumor immunotherapy is the deployment of immune checkpoint inhibitors (ICIs). These inhibitors target suppressive molecules on immune cells—for instance, PD-1 on T cells and its ligand PD-L1 on tumor cells (85, 86). By inhibiting these interactions, ICIs remove inhibitory controls on the immune system, enhancing T cells’ ability to recognize and eliminate cancer cells more effectively. Clinical research, including the KEYNOTE-240 and CheckMate-040 trials, has substantiated the effectiveness of ICIs like pembrolizumab and nivolumab in boosting antigen presentation and fostering antitumor immunity in advanced HCC cases. These treatments not only reactivate exhausted CD8+ T cells but also increase tumor-infiltrating lymphocytes (TILs) and amplify tumor antigen-specific immune responses (87–89).

Another strategy to augment antigen presentation involves cancer vaccines, which are designed to elicit a targeted immune response against tumor antigens (90). Comprising either tumor-associated antigens (TAAs) or neoantigens from mutated cancer-specific proteins, these vaccines are pivotal in initiating an immune reaction. DCs are crucial in the process of capturing, processing, and presenting antigens to T cells. Clinical trials investigating various cancer vaccine modalities, including peptide-based, protein-based, and nucleic acid-based vaccines in HCC, have demonstrated encouraging results in enhancing antigen-specific T cell responses and improving clinical outcomes. Additionally, adoptive T cell therapy (ACT) involves the ex vivo expansion and modification of patient-derived T cells to boost their antigen recognition and cytotoxic activity (91–93). Chimeric antigen receptor (CAR) T cell therapy, a subset of ACT, involves engineering T cells to express CARs that target specific tumor antigens. While CAR T cell therapy has achieved significant success in treating hematological malignancies, its application in HCC is under investigation. ACT enhances antigen presentation by directly infusing highly specific and potent T cells into the patient, counteracting tumor-induced immunosuppression and enhancing the potential for tumor eradication.

### 4.1 Overview and application prospect of cancer vaccine

The development of immunotherapy is propelled by cancer vaccines that harness the immune system’s ability to recognize and destroy tumor cells (94, 95). Specifically tailored to induce targeted immune responses against cancer-specific antigens, these vaccines activate CTLs and foster the development of immunological memory, thereby enhancing antigen presentation and triggering robust immune responses in HCC and other malignancies, which may improve clinical outcomes.

Various types of cancer vaccines exist, including peptide-based, protein-based, nucleic acid-based, and whole-cell vaccines (96, 97). Peptide-based vaccines administer short peptides derived from tumor antigens to prime T cells for cancer cell recognition and targeting. Protein-based vaccines use entire proteins or their fragments from tumor cells to provoke immune reactions. Vaccines that utilize nucleic acids deliver either DNA or RNA encoding tumor antigens, which aids in the synthesis of these proteins and stimulates the immune response. Whole-cell vaccines employ either intact tumor cells or their lysates to prompt immune recognition of a wide array of tumor antigens. At the core of these vaccination approaches are DCs, which take up, process, and present antigens to T cells, thereby initiating the immune response. In clinical settings, the use of cancer vaccines in HCC has been evaluated, such as in a phase III trial of the peptide-based vaccine adjuvant MelCancerVac, which targets melanoma-associated antigens in HCC patients. Although primary endpoints were not met, the trial underscored the potential to induce tumor-specific immune responses. Additionally, early-phase trials of the TERT-encoding DNA vaccine GRANITE-001 have yielded promising results by stimulating T cell responses against telomerase reverse transcriptase (TERT), an antigen prevalent in various cancers, including HCC.

The future of cancer vaccines in HCC appears promising but is not without its challenges. The heterogeneity of tumor antigens and immune responses among patients calls for the identification of both unique and common antigens to optimize vaccine design. Additionally, the tumor microenvironment’s immunosuppressive characteristics and possible mechanisms of immune tolerance might reduce the effectiveness of vaccines. To overcome these barriers, combination strategies, including the integration of cancer vaccines with immune checkpoint inhibitors, are under investigation to enhance therapeutic outcomes. The potential of cancer vaccines to improve antigen presentation and target specific immune responses against cancer cells underscores their role in advancing personalized immunotherapy approaches in HCC. Ongoing investigations and clinical studies are crucial for the advancement and enhancement of cancer vaccine approaches, offering the potential to greatly influence the treatment of HCC.

### 4.2 Role of CAR-T cell therapy in antigen presentation

CAR-T cell therapy, a pivotal advancement in immunotherapy, has profoundly influenced antigen presentation and the immune response to cancers, including HCC. This therapy involves T lymphocytes engineered to express chimeric antigen receptors (CARs) that merge the antigen-binding domain of an antibody with T cell signaling domains (98). These engineered receptors allow CAR-T cells to identify specific tumor-associated antigens without the need for major histocompatibility complex (MHC) presentation, circumventing conventional antigen presentation pathways. In practice, T cells are harvested from patients, genetically modified to bear CARs that target tumor-specific antigens, and then reinfused (99). These CAR-T cells then recognize and bind to tumor cells displaying these antigens, triggering direct cytotoxic attacks and cytokine release, which not only promotes tumor cell destruction but also enhances antigen presentation through direct T-cell engagement.

Research in the clinical field highlights the considerable potential of CAR-T cell therapy for the treatment of HCC. A noteworthy investigation centered on CAR-T cells targeting glypican-3 (GPC3), a unique antigen commonly found in advanced HCC cases, revealed impressive antitumor effectiveness, with several patients showing marked tumor reduction and increased survival duration (100). Additional investigations into CAR-T cells targeting other antigens like alpha-fetoprotein (AFP) and mesothelin in preclinical and early clinical phases further attest to the adaptability of this approach in HCC. However, challenges such as the immunosuppressive nature of the tumor microenvironment, tumor antigen variability, and potential antigen escape mechanisms pose hurdles to the lasting effectiveness of CAR-T cell therapy. Additionally, immune checkpoint inhibitors and CAR-T cell therapies are associated with certain adverse effects, including skin toxicity, gastrointestinal reactions, and cytokine release syndrome. To address these issues, strategies that combine CAR-T therapy with immune checkpoint inhibitors are being explored to counteract immunosuppression and enhance therapeutic results. Adverse reactions can be effectively mitigated through close monitoring, appropriate medication, and supportive care, thereby ensuring patient safety. Ongoing research into identifying optimal antigens and developing effective and safe CAR constructs is critical to refining and expanding the utility of CAR-T cell therapy in HCC treatment.

## 5 Research progress and future prospects

### 5.1 Application of new technologies in the study of immune cell function

Recent advancements have significantly enhanced our understanding of immune cells in both healthy and diseased states, including HCC. Single-cell RNA sequencing (scRNA-seq) has proven to be a powerful tool for delineating gene expression at the individual cell level (101). In HCC, scRNA-seq has exposed the diversity within immune cell populations in the tumor microenvironment, offering insights into their functional states and interactions (90). Further, developments in high-dimensional flow cytometry and mass cytometry have enabled the simultaneous measurement of multiple markers on individual cells, improving our grasp of immune cell phenotypes and functionalities. Spatial transcriptomics have also been employed to map immune cells within tumors, illuminating their spatial distribution and intercellular communication. These technological breakthroughs collectively provide a detailed picture of the dynamic immune responses and the mechanisms of immune escape in HCC, setting the stage for more targeted therapeutic approaches.

### 5.2 Development of individualized treatment strategies

The transition to personalized medicine is increasingly evident in the realm of HCC immunotherapy, underscored by the unique immune profiles and tumor characteristics exhibited by each patient (102). Detailed profiling of tumor antigens and immune cell infiltrates enhances the formulation of customized treatment plans (103). The identification of neoantigens through genomic sequencing has facilitated the creation of personalized vaccines and adoptive T cell therapies (104). Additionally, the discovery of biomarkers that predict responses to immune checkpoint inhibitors aids in selecting appropriate treatments, reducing ineffective approaches and improving patient outcomes. The combination of various omics data, such as genomic, transcriptomic, and proteomic analyses, assists in identifying the key molecular factors and pathways that contribute to immune dysfunction in HCC (105, 106). Looking ahead, the amalgamation of these insights with cutting-edge immunotherapies promises to refine clinical results by aligning interventions with the specific immunological profiles of HCC patients.

### 5.3 Directions and challenges for future research

Despite substantial progress, numerous pivotal challenges and directions remain in the domain of HCC immunotherapy. Initially, deciphering the intricate relationships between immune cells and the tumor microenvironment remains a challenging endeavor. It is crucial to comprehend the diverse functionalities of immune cells and their communication networks to devise potent combination therapies. Secondly, addressing the immunosuppression prevalent within the HCC microenvironment demands innovative approaches. The synergy of immune checkpoint inhibitors, immune modulators, and targeted therapies presents a promising route to counteract immune escape mechanisms (107). Thirdly, the enhancement of adoptive T cell therapies, such as CAR-T cells, hinges on refining engineering techniques to boost tumor specificity and longevity. Additionally, the development of universally applicable CAR-T cells or allogeneic methods could mitigate logistical and manufacturing hurdles (7). Lastly, the quest for biomarkers that can stratify patients and predict treatment responses is critical. Effective predictive markers are essential to tailor therapy choices, minimizing adverse effects while maximizing therapeutic efficacy.

## 6 Conclusion and discussion

In summary, a detailed investigation of immune cell dynamics and antigen presentation within HCC has revealed complex interaction layers that critically affect tumor progression, immune reactions, and therapeutic results. The immune evasion tactics utilized by HCC illustrate the tumor’s capacity to circumvent immune surveillance and underscore the importance of immune checkpoints like CTLA-4 and PD-1/PD-L1 in regulating the intricate equilibrium between immune activation and suppression. Various immune cells, including DCs, NK cells, and CD8+ T cells, engage in a sophisticated interaction within the tumor microenvironment. This interaction influences antigen presentation, immune infiltration, and ultimately, tumor control. The introduction of novel approaches such as cancer vaccines and CAR-T cell therapies opens promising pathways to enhance antigen presentation and empower the immune system to more effectively recognize and combat cancer cells.

Clinical investigations, such as the KEYNOTE-240 and CheckMate-040 trials, have corroborated the significant clinical advantages of immune checkpoint inhibitors in reinvigorating immune responses and enhancing outcomes for advanced HCC. Nevertheless, hurdles in patient stratification, biomarker identification, and the management of immune-related side effects persist. The integration of innovative technologies, such as single-cell RNA sequencing and high-dimensional flow cytometry, has afforded profound insights into the functionality of immune cells. This technological prowess has unraveled the complexities of immune cell populations and their interplay within HCC, thereby facilitating the crafting of personalized treatment modalities that leverage unique patient characteristics to maximize therapeutic efficacy. Furthermore, advancements in CAR-T cell therapy underscore its potential in augmenting antigen presentation, thus spearheading a new frontier in immunotherapy for HCC and potentially other malignancies.

Looking forward, the unraveling of the complex interactions between immune cells and tumors within the HCC microenvironment is imperative. This understanding is critical for the informed development of combination therapies aimed at counteracting immunosuppression. The quest for biomarkers that accurately predict treatment responses continues to be a focal point, promising to refine therapy selection to enhance patient outcomes and minimize side effects. Ongoing research is essential to perfect adoptive T cell therapies and address challenges such as immunosuppression, tumor heterogeneity, and scalability of manufacturing processes.

In summary, the comprehensive analysis of immune cell function and antigen presentation within HCC has laid a robust groundwork for innovative cancer immunotherapy strategies. The synergy of cutting-edge technologies, tailored treatment approaches, and a nuanced comprehension of the immune landscape is poised to transform the management of HCC, thereby reshaping the therapeutic paradigm for patients in dire need.
